# Neonatal Sevoflurane Exposure Exerts Sex‐Specific Effects on Cognitive Function via C3‐ and TLR4‐Related M1/M2 Microglial Cell Polarisation in Rats

**DOI:** 10.1111/jcmm.70311

**Published:** 2025-01-28

**Authors:** Jiangxia Cheng, Yuxin He, Zhuo Wang, Zhengchao Wang, Xiaohong Peng, Liangcheng Zhang

**Affiliations:** ^1^ Department of Anesthesia Fujian Medical University Union Hospital Fuzhou China; ^2^ Department of Anesthesia Wuhan Fourth Hospital Wuhan China; ^3^ Department of Orthopedics Wuhan Fourth Hospital Wuhan China

**Keywords:** Cognitive Function, Complement 3, Microglial cell, Toll‐like receptor 4

## Abstract

In this study, we aimed to explore the sex‐specific effects and mechanisms of sevoflurane exposure on the neural development of pubertal rats on the basis of M1/M2 microglial cell polarisation and related signalling pathways. A total of 48 rat pups (24 males and 24 females) were assigned to the 0‐ or 2‐h sevoflurane exposure group on the seventh day after birth. The Morris water maze (MWM) test was subsequently conducted on the 32nd to 38th days after birth. M1/M2 microglial cell polarisation, C3 and TLR4 expression, and synapse growth were analysed within specific brain zones by immunofluorescence after the MWM test. We found that the negative effects caused by sevoflurane exposure were weaker in female rats than in male rats and had less influence on spatial memory. Sevoflurane exposure has opposite effects on microglial M1 polarisation in the different sexes but can promote M2 polarisation, with more obvious effects seen in female rats. In addition, sevoflurane exposure had bidirectional effects on C3 expression in different zones, while it clearly downregulated C3 expression in female rats. Moreover, sevoflurane decreased TLR4 expression in the hippocampus, whereas female rats exhibited better resistance, especially in the dentate gyrus. Compared with male rats, female rats were more resistant to the synaptic reduction effect of sevoflurane exposure. In conclusion, we found that neonatal sevoflurane exposure could exhibit sex‐specific effects via the regulation of C3‐ and TLR4‐related microglial cell polarisation. In addition, subregional regulation in the hippocampus might also contribute to its sex‐specific effects.

## 1 Introduction

With the continuous progress of medical technology, the demand for surgery in neonates and young patients continues to rise. Moreover, an increasing number of young children in younger stages have undergone surgery under general anaesthesia. Because general anaesthesia can ensure the thoroughness of analgesia, effectively reduce tension in children and facilitate respiratory management during surgery, it has become the most common and preferred anaesthesia method for paediatric surgery [1, 2, 3]. In the past decade, animal studies have revealed that damage to neural development occurs after exposure to anaesthetics [4]. As a result, researchers have begun to pay attention to the safety of paediatric general anaesthesia [5, 6, 7].

Sevoflurane, an aryl hydrocarbon halogen inhalation anaesthetic, has gained acceptance by many young children because of its aromatic odour [8]. Because of several advantages, such as the rapid induction and awakening process, small effects on intraoperative hemodynamics, and a reduction in the amount of muscle relaxant drugs needed, sevoflurane is suitable in the general anaesthesia induction and maintenance phases for young children [9, 10]. Animal experiments and many retrospective clinical studies have shown that repeated sevoflurane exposure in children can lead to significant decreases in memory and fine motor ability in the adult stage [11, 12, 13, 14]. In addition, studies have shown that the neurotoxicity of sevoflurane is related to age, duration and hypercapnia [15, 16]. As a result, the effects of sevoflurane on the cognitive function of children need to be further explored.

Sex is an important variable in biology, and a variety of diseases show significant differences between sexes in the process of development. Similarly, sex factors also play important roles in the neural development of pubertal rats exposed to sevoflurane and its impact on cognitive function [17, 18, 19]. Our previous studies revealed that neonatal sevoflurane exposure has sex‐specific effects on cognitive function [20]. However, at present, the specific mechanism of sex differences in sevoflurane neurotoxicity is not fully understood. In addition, microglia are specialised macrophages and are important for homeostasis in the brain. M1 and M2 phenotype polarisation can significantly affect synapse and cognitive function [21, 22]. This process can be affected by multiple mechanisms, including the complement 3 (C3) and Toll‐like receptor (TLR4) pathways [23, 24].

This study aimed to explore the sex‐specific effects and mechanisms of sevoflurane exposure on the neural development of pubertal rats on the basis of M1/M2 microglial cell polarisation and its underlying mechanisms.

## 2 Methods

### 2.1 Animals

All the animal experimental protocols were reviewed and approved by the Animal Ethics Committee of Wuhan No. 4 Hospital, Wuhan, China (KY202416601). A total of 48 Sprague–Dawley rat pups (24 male and 24 female) were purchased from the Experimental Animal Center of Tongji Medical College, Huazhong University of Science and Technology. Nursing rat pups and their dams were housed, with one dam and one litter per cage, with free access to food and water. The environment was controlled on a 12‐h light/dark cycle (lights on at 08:00) at a temperature of 24°C. Nursing rat pups were raised by their dams until postnatal day 7 (P7, with P0 representing the date of birth), at which point they were randomised to two groups; at least one animal from every litter was assigned to each of the six groups. All efforts were made to minimise the number of rats used and their suffering. Male rats and female rats were analysed separately in the following experiments.

### 2.2 Neonatal Exposure to Sevoflurane

On P7, the rats were randomly allocated to one of the following protocols for 3% sevoflurane exposure: continuous exposure for 0 h or 2 h. These groups were designated male (M_0_, M_2_) and female (F_0_, F_2_), respectively. All the animals were kept in a 30% oxygen environment in an acrylic chamber within an incubator set to 37°C to maintain rectal temperatures of 36.5°C–37.5°C. The inhaled anaesthetic and oxygen concentrations were controlled by an anaesthesia apparatus (Avance CS2; General Electric, USA) and adjusted according to the instructions. Every 30 min, the pulse and peripheral oxygen saturation of the animals were measured by a handheld pulse oximeter (MD200K2; ChoiceMMed, USA). Half of the rats in each group were sacrificed using an overdose of pentobarbital for immunofluorescence detection (microglial cell polarisation detection including Iba1, C3, CD68, Arg‐1, and TLR4) after the exposure. The remaining rats were returned to their dams for further test.

### 2.3 Morris Water Maze Test

The remaining rats in each group were returned to their dams. The Morris water maze (MWM) test was used to evaluate spatial learning and memory at P32–37. Briefly, the maze consisted of a round pool (painted black, 180 cm in diameter, 60 cm in height) filled with water heated to 22°C ± 2°C. For analysis, four equal quadrants, designated I, II, III, and IV, were defined within the pool. An escape platform (8 cm in diameter) was placed at the centre of quadrant IV, approximately 1.5 cm below the water surface. The rats were subjected to four training sessions daily for five consecutive training days to locate the hidden escape platform. Each trial started from a different quadrant and was limited to 90 s. If the rat reached the platform within 90 s, the time from beginning to end was considered the escape latency. If the rat failed to find the platform in the allotted time, the escape latency was recorded as 90 s, and the rat was placed onto the platform for 20 s. On Day 6, a probe trial was performed by allowing the rat to swim for 90 s in the absence of the platform. The swimming time and trajectory of the rats were recorded by a Noldus EthoVision XT video analysis system (Noldus, Netherlands). The rats were sacrificed using an overdose of pentobarbital after the MWM test at P38 for synapse detection.

### 2.4 Immunofluorescence Detection

The brain samples were fixed in 4% paraformaldehyde for 24 h. After paraffin embedding, 4‐μm sections were obtained for immunofluorescence analysis. The rabbit primary antibodies were diluted according to the manufacturer's instructions. After deparaffinisation and rehydration, the samples were incubated overnight with diluted primary antibodies at 4°C. Fluorescein‐labelled goat secondary antibodies were added to the samples, and the samples were incubated for 1 h at room temperature. Nuclei were stained with DAPI, and the slides were scanned via an LSM 710 confocal microscope (Zeiss, Oberkochen, Germany) with an EC‐Plan‐Neofluar 40×/1.3 oil immersion objective. The quantitative data were obtained from the fluorescence intensity measurements via the ZEN 2009 software (Zeiss). The colocalisation of immunofluorescence marker was performed by ImageJ as described in previous studies [25]. The following primary antibodies were used: Iba‐1 (mouse, abcam, ab283319, 1:600), CD68 (rabbit, servicebio, GB113109, 1:300), Arg‐1 (rabbit, genetex, GTX109242, 1:300), TLR4 (rabbit, affinity, AF7017, 1:200), and C3 (rabbit, PTG, 21337‐1‐AP, 1:300), and PSD95 (rabbit, abcam, ab238135, 1:300). The following secondary antibodies were used: fluorescein‐labelled goat anti‐rabbit antibodies (proteintech, SA00013‐2, 1:100), fluorescein‐labelled goat anti‐mouse antibodies (proteintech, SA00013‐1, 1:100). All immunofluorescence staining were performed in triplicate.

### 2.5 Western Blots

The hippocampus of each rat was obtained for western blotting. The total protein content was extracted from hippocampus, and protein concentrations were determined using a BCA protein assay kit (Pierce). Total protein extract (40 g) was separated by 10% SDS–PAGE and then electrotransferred onto nitrocellulose membranes. After being blocked with TBST containing 5% skim milk, the membranes were incubated at 4°C overnight with primary antibodies (antibodies were diluted according to the manufacturer's instructions). The membranes were incubated with secondary antibodies at 37°C for 2 h (goat anti‐rabbit). After chromogen application, immunoreactive bands were obtained. Quantitative data were obtained from the results of densitometric methods by using the AlphaEaseFC software (Alpha Innotech). Rabbit anti‐cleaved C3, TLR4, CD68, and Arg‐1 primary antibodies were purchased from Abcam (Cambridge, UK). Rabbit anti‐glyceraldehyde 3‐phosphate dehydrogenase (GAPDH) and horseradish peroxidase (HRP)‐conjugated goat anti‐rabbit IgG were obtained from Proteintech (Chicago, IL, USA).

### 2.6 Real‐Time Polymerase Chain Reaction (RT‐PCR)

A total RNA kit (Takara, Japan) was used to isolate RNA from rat knee joint cartilage according to the manufacturer's instructions. An RNA PCR kit (Takara, Japan) was used to conduct reverse transcription and cDNA synthesis. The real‐time polymerase chain reaction was performed using a Step One SYBR Green Mix Kit (Takara, Japan) and an ABI Prism Sequence Detection System (Applied Biosystems, USA) according to the manufacturer's instructions. The PCR primers are shown in Table S1. All relative mRNA expression was calculated using the $-2^{{\Delta \Delta C}_{T}}$ method.

### 2.7 Statistical Analyses

Statistical analyses were performed via SPSS 21.0. The normality of the distribution was tested via a Q–Q plot. These data were analysed via repeated‐measures ANOVA. A post hoc test of the *p* value was performed with the Bonferroni correction. A value of *p* < 0.05 was considered statistically significant.

## 3 Results

### 3.1 Effects of Neonatal Sevoflurane Exposure on Cognitive Function

**FIGURE 1:**
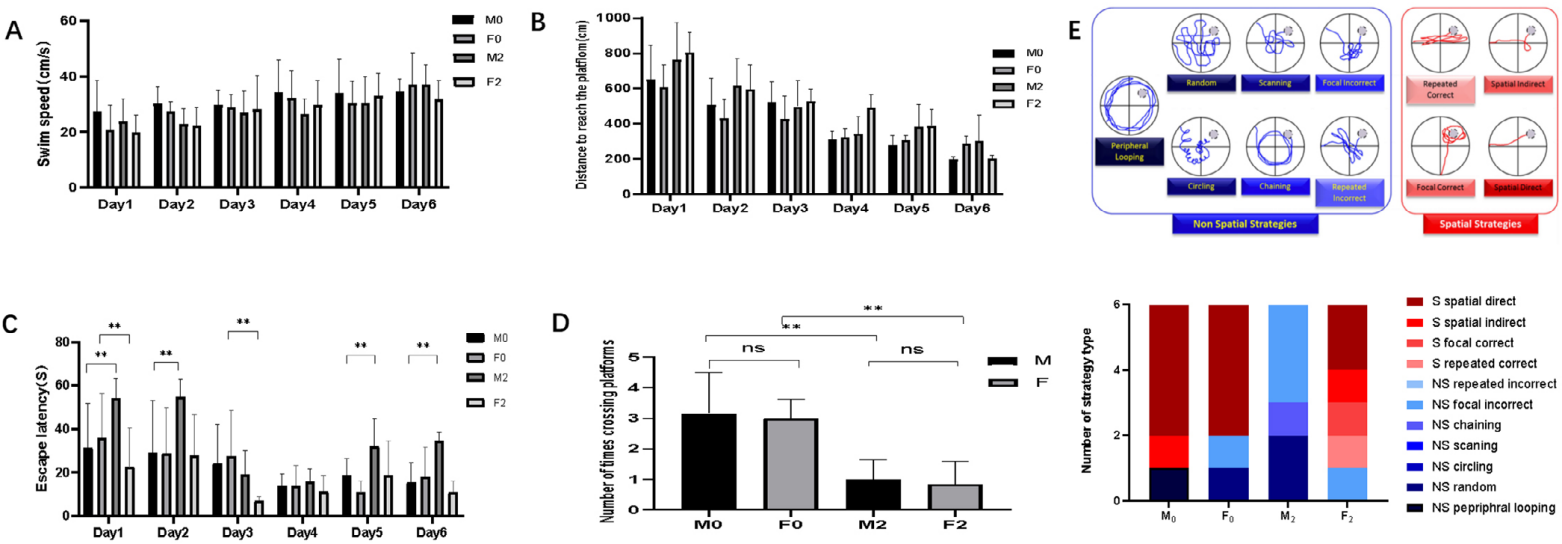
The results of Morris water maze (MWM) test. (A–D) Swim speed, distance to reach the platform, escape latency and number of platform crossings in the MWM test. (E) Navigation path analysis in the MWM test. ns, no significant difference; **p < 0.01 (M_0_—male rats without sevoflurane exposure; M_2_—male rats with 2‐h sevoflurane exposure; F_0_—male rats without sevoflurane exposure; F_2_—male rats with 2‐h sevoflurane exposure).

The effects of neonatal exposure to sevoflurane on cognitive function were assessed via the MWM test. We first compared the swim speeds of rats to eliminate bias in our analysis. As shown in Figure 1A, there were no significant differences in swimming speed among the four groups. During the spatial learning period, the rats in all the groups reached the platform with no significant differences in distance (Figure 1B). However, the escape latency of the M_2_ group was longer than that of the M_0_ group at Days 1, 2, 5 and 6, whereas that of the F_2_ group was shorter than that of the F_0_ group at Days 1 and 3 (Figure 1C). Moreover, the number of platform crossings in both the M_2_ and F_2_ groups was lower than that in the M_0_ and F_0_ groups (Figure 1D, *p* < 0.01).

To further explore the effects of neonatal exposure to sevoflurane on spatial memory, we analysed the navigation path during the MWM test as described in a previous study [26]. As shown in Figure 1E, spatial strategies were predominant in both the M_0_ and F_0_ groups. However, after 2 h of neonatal sevoflurane exposure, nonspatial strategies were predominant in the M_2_ group, whereas spatial strategies were still predominant in the F_2_ group. These results demonstrate that neonatal exposure to sevoflurane can negatively affect cognitive function in both male and female rats. However, the negative effects were weaker for female rats, with less influence on spatial memory.

### 3.2 The Effects of Neonatal Sevoflurane Exposure on Microglial Cell Activation and Polarisation in the Hippocampus

**FIGURE 2:**
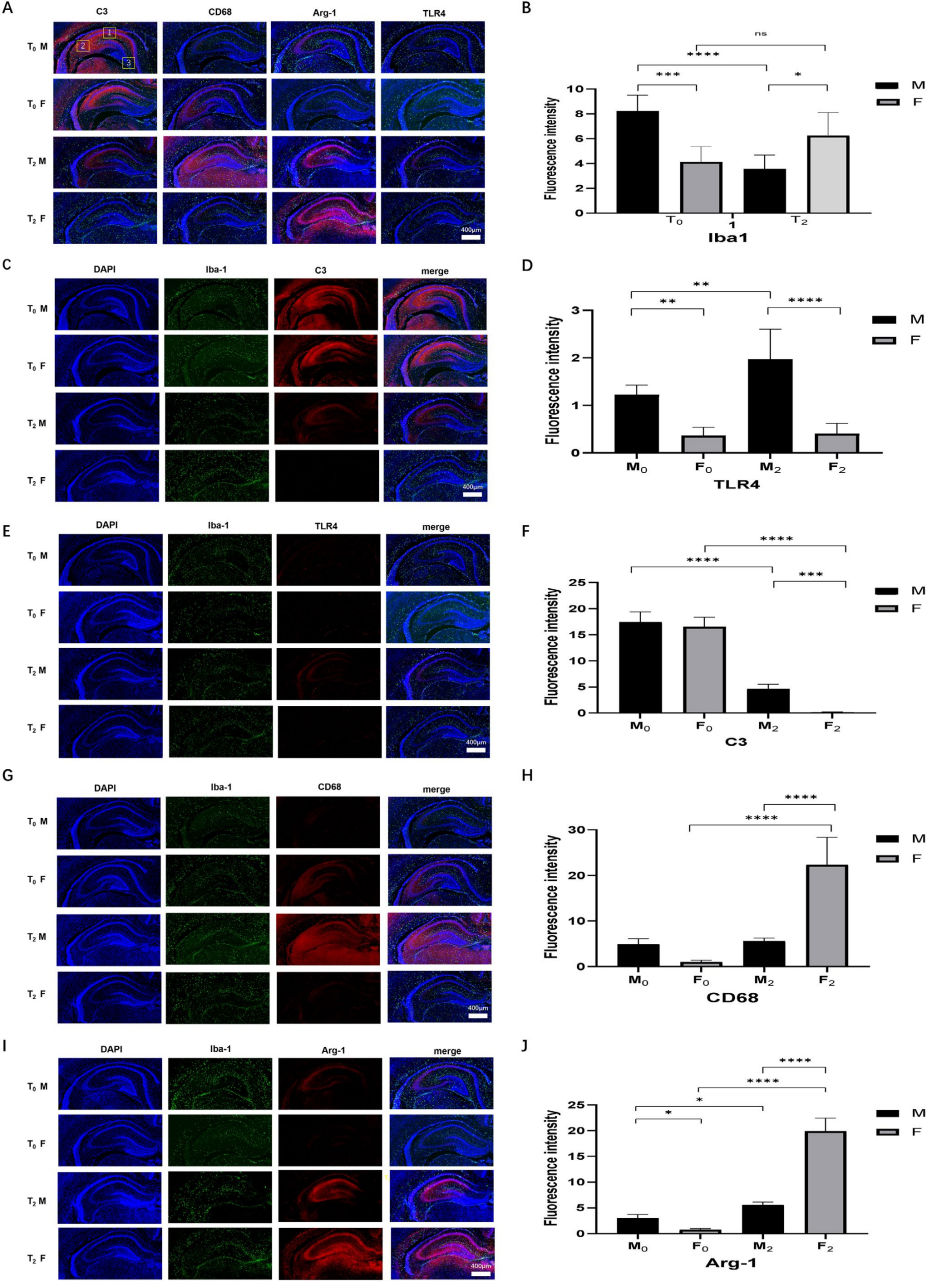
Effect of neonatal sevoflurane exposure on microglial activation and C3, TLR4, CD68 and Arg‐1 in hippocampus detected by immunofluorescence. (A) The double fluorescent merged staining image of C3, CD68, Arg‐1 and TLR4. □1 represents CA1 zone; □2 represents CA3 zone, □3 represents DG zone. (B) The fluorescence intensity of Iba1 in hippocampus. (C) The double fluorescent staining image of Iba1 and C3. (D) The fluorescence intensity of C3 in hippocampus. (E) The double fluorescent staining image of Iba1 and Arg‐1. (F) The fluorescence intensity of TLR4 in hippocampus. (G) The double fluorescent staining image of Iba1 and TLR‐4. (H) The fluorescence intensity of CD68 in hippocampus. (I) The double fluorescent staining image of Iba1 and CD68. (J) The fluorescence intensity of Arg‐1 in hippocampus. *p < 0.05; **p < 0.01; ***p < 0.005; ****p < 0.001 (T0—control group without sevoflurane exposure; T2—group with 2‐h sevoflurane exposure; M—male rats; F—female rats).

The effects of neonatal exposure on microglial cell activation and polarisation in the hippocampus were determined by immunofluorescence. Iba 1 expression is a biomarker of activated microglia. CD68 expression is a biomarker of M1 microglia, whereas Arg‐1 expression is a biomarker of M2 microglia. Compared with that in the F_0_ group, microglial activation in the whole hippocampus was greater in the M_0_ group (Figure 2B, *p* < 0.001). However, after sevoflurane exposure, microglial cell activation in male rats decreased (*p* < 0.001), whereas that in female rats changed little (*p* > 0.05, Figure 2B). These results suggest that sevoflurane has opposite effects on microglial cell activation in the different sexes.

We first detected the expression levels of C3, TLR4, CD68, and Arg‐1 in the whole hippocampus using immunofluorescence, western blot and RT‐PCR. As shown in Figure 2C–K, Figures S1 and S2, female rats exhibited relatively lower levels of all these proteins than male rats. After a 2‐h sevoflurane exposure, TLR4 (*p* < 0.001), CD68 (*p* < 0.05) and Arg‐1 (*p* < 0.001) levels upregulated, while TLR4 levels downregulated (*p* < 0.001) in male rats. In female rats, C3 and TLR4 levels downregulated (*p* < 0.001), while CD68 and Arg‐1 levels upregulated (*p* < 0.001) after the 2‐h sevoflurane exposure.

**FIGURE 3:**
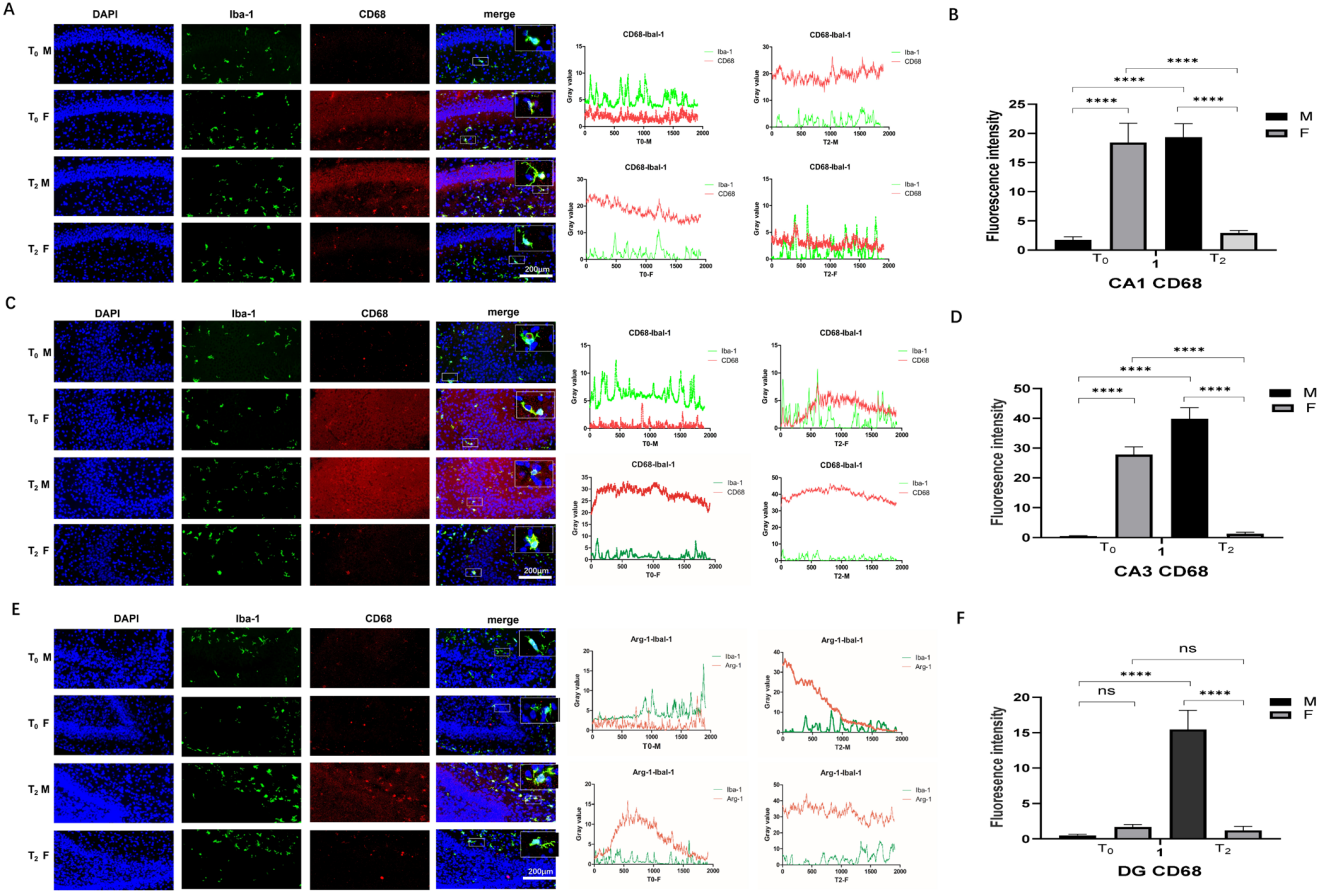
Effect of neonatal sevoflurane exposure on M1 microglial polarisation in the hippocampus. (A) The double fluorescent staining image and grey value analysis of Iba1 and CD68 in CA1. (B) The fluorescence intensity of CD68 expression in CA1. (C) The double fluorescent staining image and grey value analysis of Iba1 and CD68 in CA3. (D) The fluorescence intensity of CD68 expression in CA3. (E) The double fluorescent staining image and grey value analysis of Iba1 and CD68 in DG. (F) The fluorescence intensity of CD68 expression in the DG. ns, no significant difference. ****p < 0.001 (T0—control group without sevoflurane exposure; T2—group with 2‐h sevoflurane exposure; M—male rats; F—female rats).

The expression of CD68 (M1 microglia) was different in different zones of the hippocampus. In CA1 and CA3, the F_0_ group presented higher CD68 expression than the M_0_ group did (*p* < 0.001, Figure 3A–D). After sevoflurane exposure, CD68 expression was increased in male rats (*p* < 0.001) but decreased in female rats (*p* < 0.001, Figure 3A–D). However, in the dentate gyrus (DG), there were no significant differences among the M_0_, F_0_ and F_2_ groups (*p* > 0.05, Figure 3E,F). The M2 group presented increased CD68 expression in the DG (*p* < 0.001, Figure 3E,F). These results suggest that sevoflurane has opposite effects on microglial M1 polarisation in the different sexes.

**FIGURE 4:**
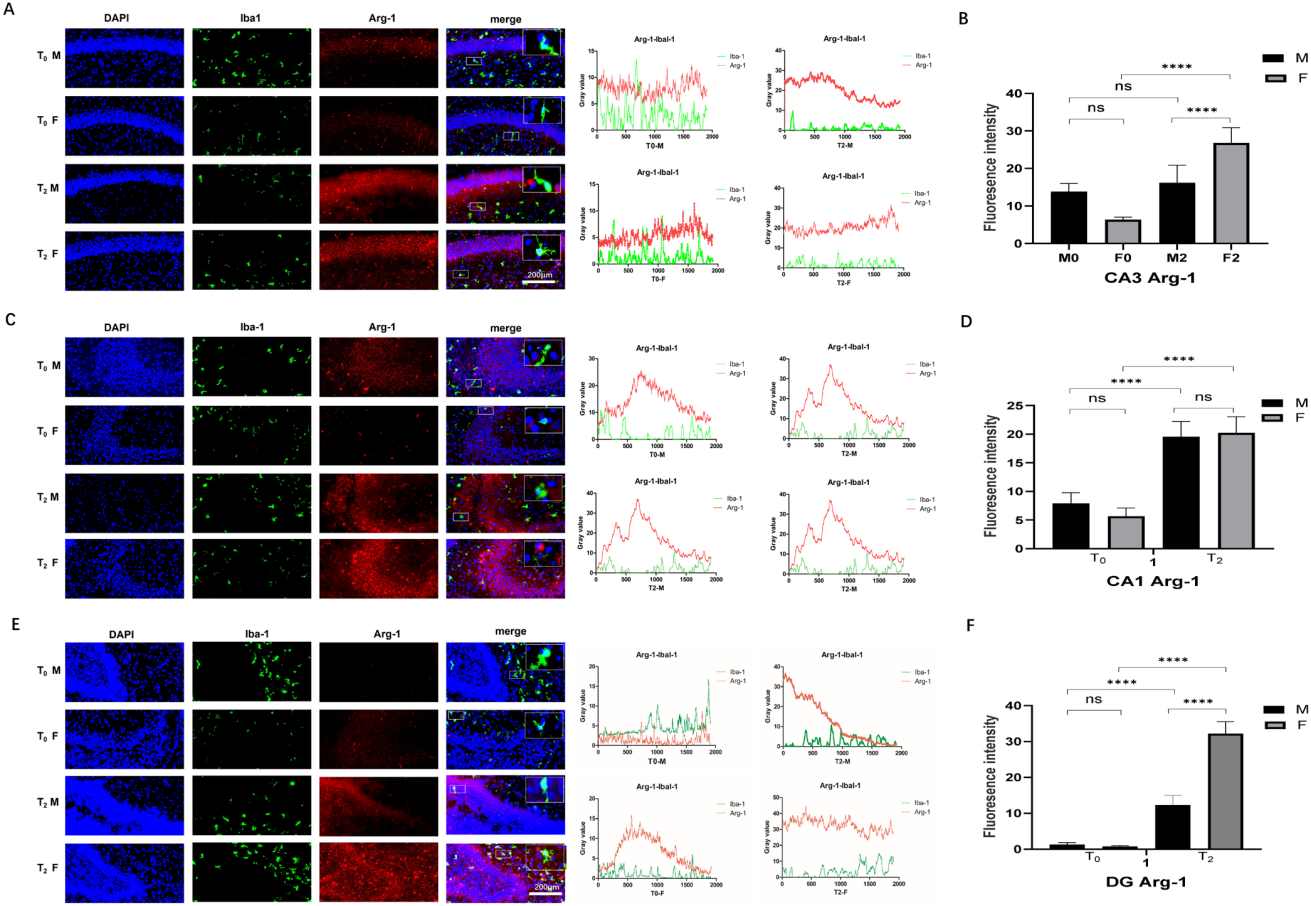
Effect of neonatal sevoflurane exposure on M2 microglial polarisation in the hippocampus. (A) The double fluorescent staining image and grey value analysis of Iba1 and Arg‐1 in CA1. (B) The fluorescence intensity of Arg‐1 expression in CA1. (C) The double fluorescent staining image and grey value analysis of Iba1 and Arg‐1 in CA3. (D) The fluorescence intensity of Arg‐1 expression in CA3. (E) The double fluorescent staining image and grey value analysis of Iba1 and Arg‐1 in DG. (F) The fluorescence intensity of Arg‐1 expression in the DG. ns, no significant difference. ****p < 0.001 (T0—control group without sevoflurane exposure; T2—group with 2‐h sevoflurane exposure; M—male rats; F—female rats).

The expression of Arg‐1 (M2 microglia) in the CA1, CA3 and DG was similar between the M_0_ and F_0_ groups. After sevoflurane exposure, Arg‐1 expression was increased in all three zones in both male and female rats (*p* < 0.001, Figure 4). However, in CA1, Arg‐1 expression upregulation was significant in both male and female rats (*p* < 0.001, Figure 4A,B). Compared with male rats, female rats presented more obvious Arg‐1 expression upregulation in the CA3 region and DG (*p* < 0.001, Figure 4C–F). These results suggest that sevoflurane exposure promoted microglial cell M2 polarisation, which was more obvious in female rats than in male rats.

### 3.3 Effects of Neonatal Sevoflurane Exposure on C3 and TLR4 Expression in the Hippocampus

**FIGURE 5:**
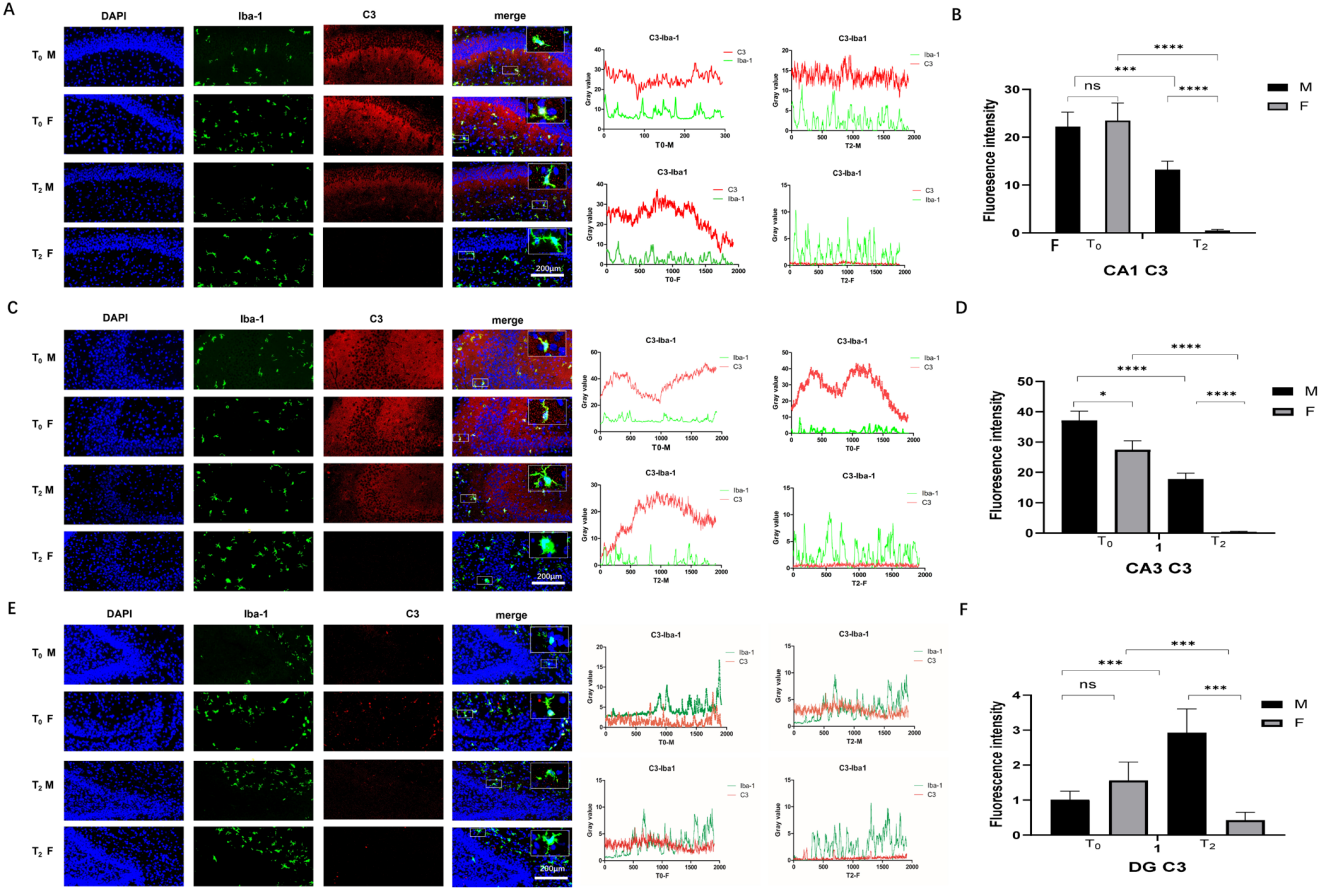
Effect of neonatal sevoflurane exposure on C3 expression in the hippocampus. (A) The double fluorescent staining image and grey value analysis of Iba1 and C3 in CA1. (B) The fluorescence intensity of C3 expression in CA1. (C) The double fluorescent staining image and grey value analysis of Iba1 and C3 in CA3. (D) The fluorescence intensity of C3 expression in CA3. (E) The double fluorescent staining image and grey value analysis of Iba1 and C3 in DG. (F) The fluorescence intensity of C3 expression in the DG. ns, no significant difference. *p < 0.05; ***p < 0.005; ****p < 0.001 (T0—control group without sevoflurane exposure; T2—group with 2‐h sevoflurane exposure; M—male rats; F—female rats).

C3 expression is crucial for the dynamic regulation of neuronal synaptic pruning by microglia [27, 28]. Moreover, TLR4 expression can accelerate the migration of microglia, promote their activation, increase their phagocytic efficiency and promote their proliferation. C3 and TLR4 expressions in the hippocampus were determined by immunofluorescence. As shown in Figure 5A–D, in CA1 and CA3, neonatal sevoflurane exposure downregulated C3 expression in both male and female rats (*p* < 0.005). However, this effect is much stronger in female rats than in male rats. With respect to the DG, neonatal sevoflurane exposure caused the expression of C3 to be upregulated in male (*p* < 0.005) rats but still downregulated it in female rats (*p* < 0.005). These results suggest that in male rats, sevoflurane has bidirectional effects on C3 expression in different zones, whereas it has obvious downregulatory effects on C3 expression in female rats.

**FIGURE 6:**
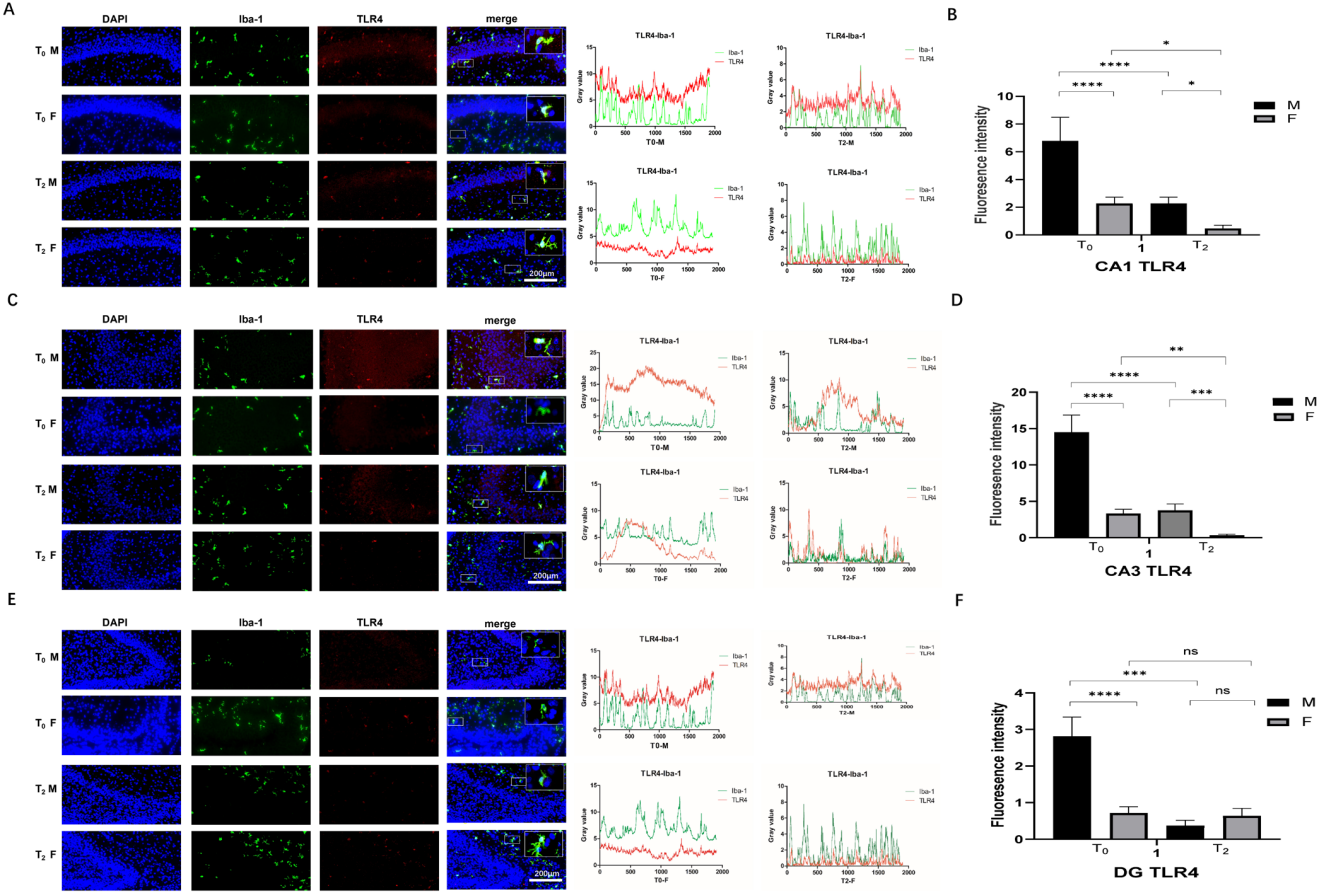
Effect of neonatal sevoflurane exposure on TLR4 expression in the hippocampus. (A) The double fluorescent staining image and grey value analysis of Iba1 and TLR4 in CA1. (B) The fluorescence intensity of TLR4 expression in CA1. (C) The double fluorescent staining image and grey value analysis of Iba1 and TLR4 in CA3. (D) The fluorescence intensity of TLR expression in CA3. (E) The double fluorescent staining image and grey value analysis of Iba1 and TLR in DG. (F) The fluorescence intensity of TLR expression in the DG. ns, no significant difference. *p < 0.05; **p < 0.01; ***p < 0.005; ****p < 0.001 (T0—control group without sevoflurane exposure; T2—group with 2‐h sevoflurane exposure; M—male rats; F—female rats).

Neonatal sevoflurane exposure also had obvious sex‐ and zone‐specific effects on TLR4 expression. As shown in Figure 6A–D, male rats presented relatively high TLR4 expression. After sevoflurane exposure, TLR4 expression was decreased in both male and female rats, whereas these effects were more obvious in male rats (*p* < 0.001) than in female rats (*p* < 0.05, Figure 6A–D). As for DG zone, compared with female rats, male rats still presented relatively high TLR4 expression (*p* < 0.001, Figure 6E,F). After sevoflurane exposure, TLR4 expression was significantly decreased in male rats (*p* < 0.005) but not in female rats (*p* > 0.05, Figure 6E,F). These results suggest that sevoflurane can downregulate TLR4 expression in the hippocampus, whereas female rats exhibit greater resistance, especially in the DG.

### 3.4 Effects of Neonatal Sevoflurane Exposure on Synaptogenesis in the Hippocampus

**FIGURE 7:**
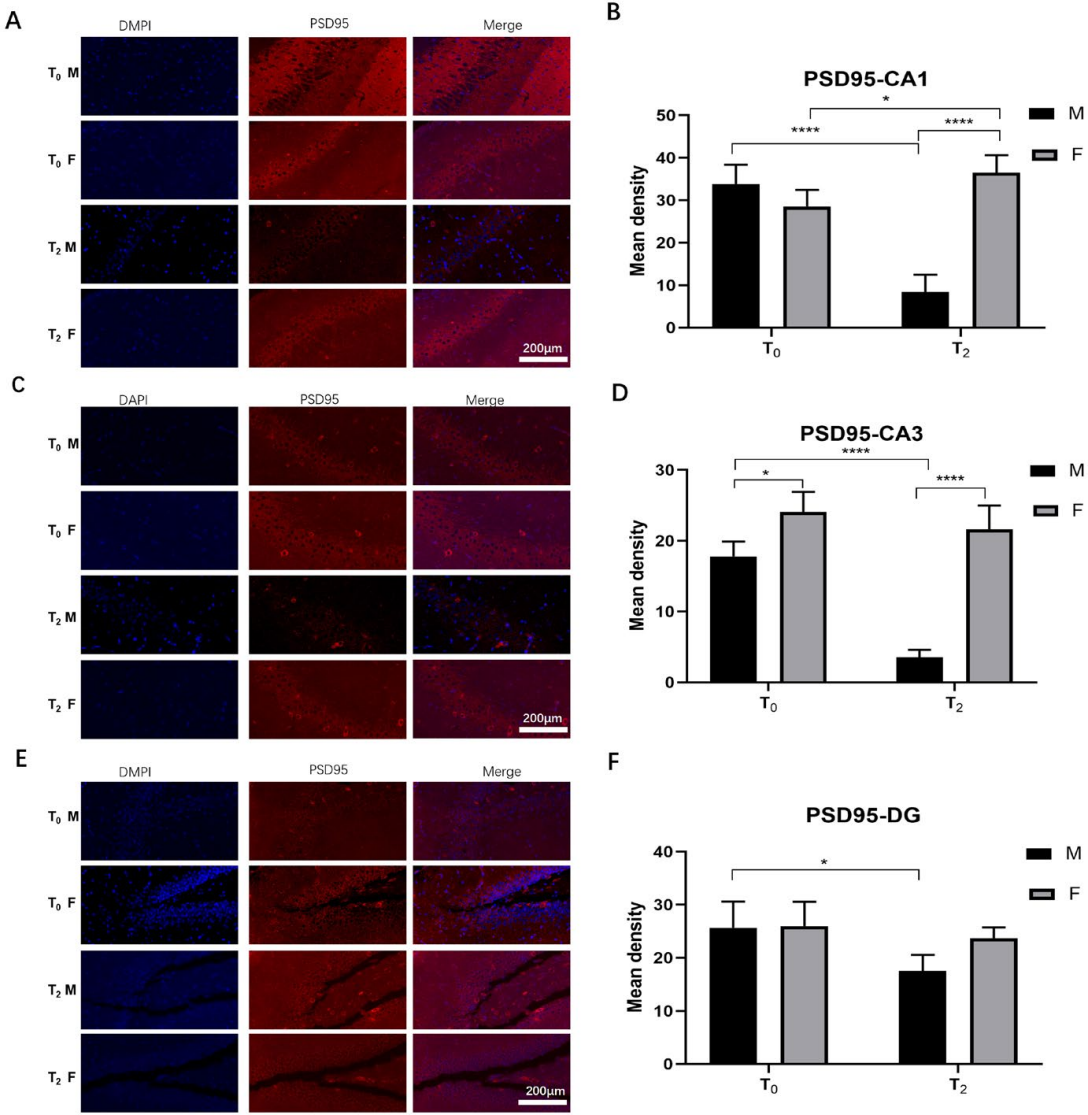
Effect of neonatal sevoflurane exposure on synapses in the hippocampus. (A, B) The fluorescence intensity of PSD95 expression in CA1. (C, D) The fluorescence intensity of PSD95 expression in CA3. (E, F) The fluorescence intensity of PSD95 expression in the DG. ns, no significant difference; *p < 0.05; ****p < 0.001 (T0—control group without sevoflurane exposure; T2—group with 2‐h sevoflurane exposure; M—male rats; F—female rats).

The effects of neonatal exposure on synaptogenesis were determined by PSD95 immunofluorescence. As shown in Figure 7, sevoflurane decreased PSD95 expression in male rats in all three zones (CA1: *p* < 0.005; CA3: *p* < 0.001; DG: *p* < 0.05). However, it had almost no effect on PSD95 expression in female rats (CA1: *p* < 0.05; CA3: *p* < 0.01; DG: *p* > 0.05). These effects suggest that female rats are more resistant to the synaptic reduction effect of sevoflurane exposure.

## 4 Discussion

Previous studies have shown that sevoflurane exposure can have a negative effect on cognitive function after 2 h but has unknown sex‐specific effects [20]. On the basis of our previous research, we further explored the sex‐specific effects of 2 h of exposure. Our results revealed that female rats exhibited better tolerance with shorter escape latency than male rats did, which further supported our hypothesis. Using the MWM test, we analysed navigation to determine spatial memory. Our results revealed that female rats exhibit better tolerance to neonatal sevoflurane exposure with fewer changes in spatial strategies, which could be a possible reason for their improved escape latency compared with males. However, our previous study revealed that neonatal sevoflurane has duration‐dependent effects. We focused on 2‐h exposure because it has the most obvious effects on cognitive function. The duration‐dependent effects need to be further explored in future.

Previous studies have shown that the classic complement cascade is a ‘tagging’ mechanism for pruning microglia [29]. One of the most significant complements, C3, is produced mainly by astrocytes and microglia. The combination of C3 and C3 receptors could result in synaptic devour [29, 30]. The inhibition of C3 activity could attenuate the loss of synapses, which might benefit cognitive function [30]. However, studies have also revealed that knockout of C3 or C3 receptor expression can cause defects in synapse clips and connections [31, 32]. Previous studies have shown that sevoflurane exposure can decrease the plasma C3 level in rats [33]. In our study, we found that after neonatal sevoflurane exposure, C3 expression was decreased in the CA1 and CA3 zones. In addition, the expression in female rats decreased more than that in male rats. In the DG, sevoflurane caused C3 expression upregulation in male rats and C3 expression downregulation in female rats, which could partially explain the differences in changes in cognitive function.

TLR4, an important member of the Toll‐like receptor family, can lead to neuroinflammation during brain injury in neonatal rats [34]. The activation of the TLR4 signalling pathway can lead to neuroinflammation via both MyD88‐dependent and MyD88‐independent pathways [35]. It can also lead to the apoptosis and pyroptosis of neurocytes, resulting in cognitive dysfunction [36]. Andreas et al. noted that the administration of a combination of TLR4 and complement could be a potential treatment strategy for cognitive dysfunction [37]. In our research, we determined TLR4 expression. In CA1 and CA3, the expression was similar to that in C3, while the male rats had higher TLR4 expression before sevoflurane exposure. However, in the DG, female rats presented resistance to decreased TLR4 expression, similar to that of C3 expression upregulation in male rats. The different changes in the DG zone could be another reason for the sex‐specific effects of sevoflurane. However, further studies need to be conducted to explore the underlying mechanisms involved.

Microglia are specialised macrophages in the brain that are important for homeostasis in the brain and synapses. Like other macrophages in other systems, microglia can also be divided into the M1 phenotype (classic activation) and the M2 phenotype (alternative activation) [38]. Some researchers regard microglia in the resting state as the M0 phenotype [39]. The M1 phenotype can exhibit proinflammatory effects, whereas the M2 phenotype can exhibit anti‐inflammatory effects [40]. When the microenvironment in the brain is affected by infection, injury or other stimulating factors, the phenotypes of microglia can change [40]. In our study, we detected the M1 phenotype using CD68 expression and the M2 phenotype via Arg‐1 expression in the hippocampus. The results revealed that, with respect to M1 polarisation, neonatal sevoflurane could increase the level of M1 polarisation in male rats and decrease the level of M1 polarisation in female rats, although the baseline levels of M1 polarisation differed. In addition, sevoflurane upregulated M2 polarisation in both male and female rats, and it was more prominent in female rats. Published studies have shown that the inhibition of C3 and TLR4 expression could increase M2 polarisation and decrease M1 polarisation in microglia [23, 41]. Our results were essentially consistent with those of previous studies. However, the increase in M1 polarisation cannot be explained by C3 and TLR4 expression inhibition, which needs to be further explored.

Although the CA1 and CA3 areas can autonomously control memory formation under certain conditions, the DG still has the most important effects on integrative memory support and spatial memory in the hippocampus [42, 43]. Another published study revealed that neonatal sevoflurane exposure improved performance in a DG‐dependent learning task in rats [44]. In our research, especially in terms of zone‐specific detection, the DG often exhibits different changes than the CA1 and CA3. In addition, the differences between males and females were more apparent in the DG than in the CA1 or CA3. These results indicate that the different changes in the DG could be a possible reason for the sex‐specific effects of neonatal sevoflurane exposure. However, the exact functions and connections of the DG, CA1 and CA3 in cognitive function and memory are still unclear. Whether and how DG changes contribute to the sex‐specific effects of sevoflurane also need to be further explored.

Sevoflurane is generally suggested to be a neurotoxic drug that can harm cognitive function and synapses. However, published studies have also revealed that sevoflurane has neuroprotective effects [44]. Our previous studies revealed that neonatal sevoflurane exposure for a certain duration could promote synapse generation and partially improve cognitive function [20]. In this study, we found that after 2 h of neonatal exposure to sevoflurane, cognitive function changed in opposite manners in male and female rats. The synapses detected by PSD95 immunofluorescence were consistent with the results of the MWM test. In male rats, the number of synapses decreased after sevoflurane exposure, whereas it did not change substantially in female rats. On the basis of our results, we suspect that changes in C3‐ and TLR4‐related M1/M2 microglial polarisation could be self‐protective effects after sevoflurane exposure. Moreover, this effect was stronger in female rats. However, the details of the underlying mechanisms are still unknown and need to be further explored.

Sevoflurane is widely used in paediatric anaesthetic drugs, and its effects on cognitive impairment and neurological development have been the focus of anaesthesiology and neuroscience. However, whether sevoflurane exposure has positive or negative effects on cognitive function and neural development is still controversial. In addition, a published study revealed that sex hormones promote safety during sevoflurane exposure by ameliorating systemic inflammation, indicating the underlying crosstalk among sex hormones, sevoflurane and cognitive function [45]. Although our research cannot determine the exact effects and mechanisms of sevoflurane on cognitive function and neurological development, the sex‐specific and subregional analysis results provide a new perspective for understanding the safety and application of sevoflurane in paediatric anaesthesia. Moreover, this study provides important information when the sex of an animal is chosen for further sevoflurane‐related studies. In conclusion, we found that neonatal sevoflurane could exhibit sex‐specific effects via the regulation of C3‐ and TLR4‐related microglial cell polarisation. In addition, subregional regulation in the hippocampus might also contribute to its sex‐specific effects.

## Author Contributions

**Jiangxia Cheng:** conceptualization (equal), data curation (equal), funding acquisition (equal), methodology (equal), project administration (equal), software (equal), validation (equal), writing – original draft (equal). **Yuxin He:** conceptualization (equal), data curation (equal), investigation (equal), software (equal), writing – original draft (equal). **Zhuo Wang:** formal analysis (equal), software (equal), validation (equal). **Zhengchao Wang:** conceptualization (equal), methodology (equal), writing – original draft (equal). **Xiaohong Peng:** funding acquisition (equal), investigation (equal), project administration (equal), software (equal), writing – review and editing (equal). **Liangcheng Zhang:** project administration (equal), supervision (equal), writing – review and editing (equal).

## Ethics Statement

All experimental animals were maintained in accordance with the Guide for the Care and Use of Laboratory Animals of the National Institutes of Health and were approved by the Ethics Committee for Animal Experimentation of Wuhan University.

## Consent

The authors have nothing to report.

## Conflicts of Interest

The authors declare no conflicts of interest.

## Supporting information


Figure S1.

Figure S2.

Table S1.

## Data Availability

The data support the findings of this study are available from the corresponding author upon reasonable request.
