# Supplementary figures and images for: Neonatal Sevoflurane Exposure Exerts Sex‐Specific Effects on Cognitive Function via C3‐ and TLR4‐Related M1/M2 Microglial Cell Polarisation in Rats
Source: J Cell Mol Med. 2025 Jan 28;29(2):e70311. doi: 10.1111/jcmm.70311 (PMC11774238; doi:10.1111/jcmm.70311)

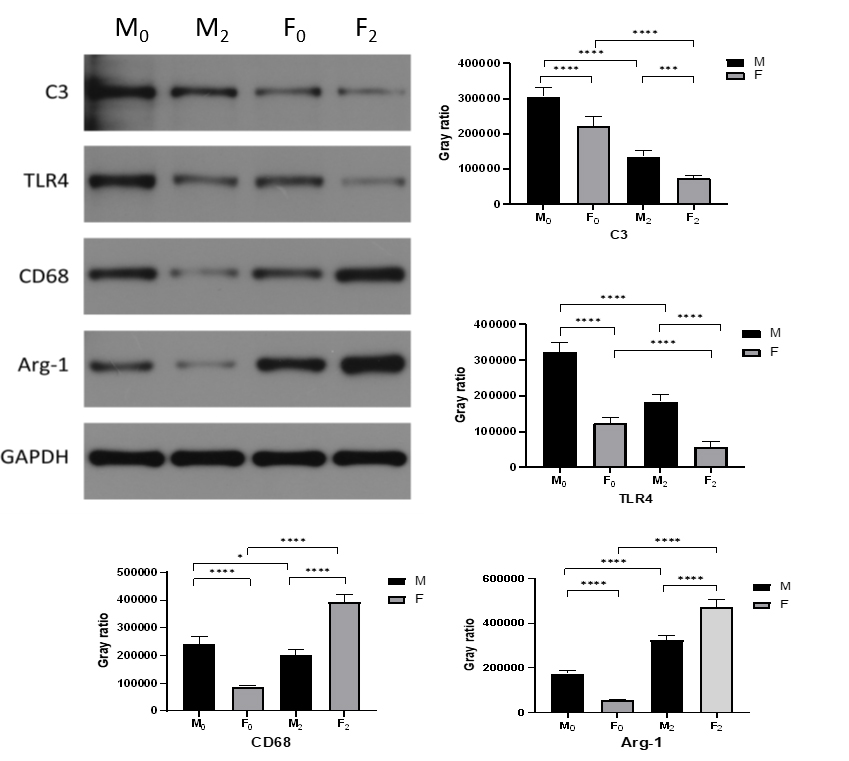

Supplement: Supplementary file 1 — Figure S1. [file JCMM-29-e70311-s001.jpg]

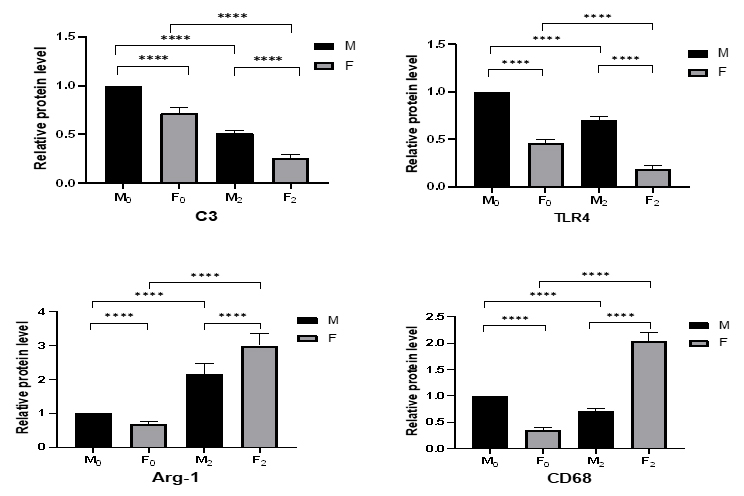

Supplement: Supplementary file 2 — Figure S2. [file JCMM-29-e70311-s002.jpg]
