# Supplementary material for: Neonatal Sevoflurane Exposure Exerts Sex‐Specific Effects on Cognitive Function via C3‐ and TLR4‐Related M1/M2 Microglial Cell Polarisation in Rats
Source: J Cell Mol Med. 2025 Jan 28;29(2):e70311. doi: 10.1111/jcmm.70311 (PMC11774238; doi:10.1111/jcmm.70311)
Supplement: Supplementary file 3 — Table S1. [file JCMM-29-e70311-s003.docx]

Figure S1. Effect of neonatal sevoflurane exposure on C3, TLR4, CD68, and Arg-1 in hippocampus detected by western blot. *, p<0.05; **, p<0.01; ***, p<0.005; ****, p<0.001. (T0-cotrol group without sevoflurane exposure; T2-group with 2-hour sevoflurane exposure; M-male rats; F-female rats)

Figure S2. Effect of neonatal sevoflurane exposure on C3, TLR4, CD68, and Arg-1 in hippocampus detected by RT-PCR. *, p<0.05; **, p<0.01; ***, p<0.005; ****, p<0.001. (T0-cotrol group without sevoflurane exposure; T2-group with 2-hour sevoflurane exposure; M-male rats; F-female rats)

Table S1 Primer sequence of RT-PCR

| Gene | Sequence (5’ -3’) |
| --- | --- |
| C3 | F: 5’- ACTACCTACACTTGTCAGTGTCACG-3’ |
|  | R: 5’-AGTCTTCTGGGTGGCAGTGAT-3’ |
| TLR4 | F: 5’- ACACTTTATTCAGAGCCGTTGGT-3’ |
|  | R: 5’- CAGGTCCAAGTTGCCGTTTC-3’ |
| CD68 | F: 5’-ATTTCGCTTCCACATTTCCTAC-3’ |
|  | R: 5’-TTGTAGAGTGAATCCAGTTATGGG-3’ |
| Arg-1 | F: 5’-ACACGGCAGTGGCTTTAACC-3’ |
|  | R: 5’-GGCGTTTGCTTAGTTCTGTCTG-3’ |
